# Camouflage or Coincidence? Investigating the Effects of Spatial and Temporal Environmental Features on Feral Cat Morphology in Tasmania

**DOI:** 10.1002/ece3.70530

**Published:** 2024-11-11

**Authors:** Alexandra J. Paton, Barry W. Brook, Jessie C. Buettel

**Affiliations:** ^1^ School of Natural Sciences University of Tasmania Hobart Tasmania Australia; ^2^ ARC Centre of Excellence for Australian Biodiversity and Heritage (CABAH) University of Tasmania Hobart Tasmania Australia

**Keywords:** adaptation, camouflage, fur, genetics, lunar phase, pelt, trail camera

## Abstract

Variations in coat morphology are well documented among felids and are theorised to aid in camouflage during stalk and ambush hunting. A diverse array of coat types has arisen in *Felis catus* (feral cats) through domestication and subsequent selective breeding. This species has successfully spread across Australia over the past 200 years, raising the question of whether any specific coat types offer an adaptive advantage. We used 24,657 camera‐trap images of feral cats in Tasmania, Australia, and assigned each cat observation a coat colour and pattern. We analysed these data to examine how different spatial features affect the modal coat type present at a site. We also tested if cats with differing coat types were active on different days in response to temporal features, including moon luminosity (full or new). Elevation was positively associated with the presence of orange (odds ratio = 2.5, 97.5% confidence interval = 1.5, 4.4) and tortoiseshell (odds ratio = 4.1, CI = 1.6, 10.5) cats, while blotched brown cats were negatively associated with elevation (odds ratio = 0.64, CI = 0.5, 0.9), relative to black cats. Brown mackerel cats were more common in eucalypt and rainforests (odds ratio = 1.9, CI = 1.1, 3.3), as well as sites with a higher FPAR (odds ratio = 1.3, CI = 1.1, 1.6). All coat types were 1.2–2 times more likely to be active on nights with a new moon, except for orange cats who were equally active regardless of moon luminosity (odds ratio = 0.94, CI = 0.62, 1.42). Our results indicate that coat types are equally successful across Tasmania, perhaps owing to naïve prey or limited predator competition. The high activity of orange cats irrespective of moon phase may be reflective of the male cat's tendency to patrol territory, as opposed to favouring dark nights for hunting. Future studies should consider comparing the coat types found in feral cats to adjacent domestic populations, and against a wider array of habitat types to further investigate the potential for selective pressure on feral cat coat types in Australia.

## Graphical abstract

Feral cats (*Felis catus*) have spread across Australia over the past 200 years, displaying various coat types, yet it remains unknown if any offers an adaptive advantage. Using a dataset of 24,657 camera‐trap images, we examined if any coat types correlate with spatial or temporal features. We found little evidence that coat type occurrence relates to spatial features within Tasmania. However, we observed that orange cats were the only coat type not to increase their activity on nights with new moons, likely owing to their sex‐linked bias towards being males and subsequent tendency to patrol territory rather than just hunt.

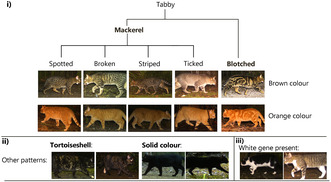

## 1 Introduction

Domestic cats (*Felis catus*) were brought to Australia over 200 years ago (Koch, Algar, and Schwenk 2016), and now occupy over 99% of the continent (Legge et al. 2017). This species was domesticated from *Felis lybica*, the African wildcat (Driscoll et al. 2007), a felid with sandy‐grey fur and vertical stripes (Werdelin and Olsson 1997) well suited for its grassy, arid origins. Selective breeding over 9000 years (Driscoll et al. 2007) has resulted in diverse coat types in domestic cat populations, including blotches, ticks, spots, stripes, solid black and ‘broken’ patterns (Eizirik et al. 2010). Although a variety of coat patterns and colours persist in feral populations (Jones and Horton 1984; Yip et al. 2014), it remains unknown whether these genetic differences confer adaptive advantages to wild cats in Australia.

There are several evolutionary advantages coat pattern and colour could provide. Generally speaking, felid species tend to stalk and ambush prey (Hopcraft, Sinclair, and Packer 2005; McGregor et al. 2015), a method which relies on a cat remaining undetected by its target. It is hypothesised that the repeated and relatively rapid evolution of diverse coat types across multiple felid species is due to their benefit in improving camouflage across different spatial features (Allen et al. 2011). For example, felid species with relatively uniform flanks are generally found in uniformly textured environments, while forest‐dwelling felids have spots to blend in with the dappled light emerging through the canopy (Allen et al. 2011).

This camouflage can also be adapted to provide an advantage in hunting across temporal environmental features. Some species of felid display a melanistic morph (entirely black) as well as patterned individuals (spotted or striped) within the same population (Werdelin and Olsson 1997). This is true for populations of feral cats in Australia (Jones and Horton 1984), and is a common trait in felid populations that are both diurnally and nocturnally active (rather than only diurnal or nocturnal) (Allen et al. 2011). Disruptive selection (also known as diversifying selection) has been suggested as the underlying mechanism for this phenomenon (Allen et al. 2011). This occurs when extreme differences in traits within a population are favoured over intermediate traits. It is hypothesised that melanistic and patterned individuals use the environment differently to reduce intraspecific competition. For example, Graipel et al. (2014) demonstrated that within a population of oncillas (*Leopardus tigrinus*), melanistic individuals were more active on bright nights, while spotted oncillas had higher activity on dark nights. They theorised that the difference in activity between coat types may have reduced intraspecific competition for hunting grounds. Considering the extreme differences in coat types present within feral cat populations, it is possible that these populations have adapted to partition resources across temporal features in a similar fashion.

Koch, Algar, and Schwenk (2016) identified significant allele frequency differences between feral and stray/house cats, indicating that feral cats in Australia are genetically isolated from domestic populations. This suggests the potential for natural selection to occur within feral populations with minimal influence of immigration from domestically bred cats. With a generation length of approximately 2.7 years (calculated from Pacifici et al. 2013; using data from Warner 1985; van Aarde 1983; Ng, Fascetti, and Larsen 2023), roughly 75 generations of feral cats have emerged in Australia over 200 years. Whether this is enough time for phenotypic changes to occur will depend on the strength of the selective pressures faced by feral cats. It has been anecdotally suggested that camouflage drives coat occurrence in arid Australia (Read and Bowen 2001), though not formally tested. Alternatively, Australia's predator‐naive prey may limit the benefits of camouflage for feral cats (Ross et al. 2019). Understanding if and how feral cat phenotypes have adapted to the Australian landscape will require disentangling the influence of spatial and temporal features on cat occurrence.

Our primary aim is to map the distribution of feral cat coats in Tasmania, Australia, utilising images from an existing long‐term camera‐trap network. We explore spatial variations in the presence of different coat types at different camera sites, considering factors such as elevation, vegetation type, photosynthetically active radiation (FPAR) and topographic roughness. To understand if cats of each morph exhibit distinct activity patterns, we analyse the influence of temporal factors, including temperature, rainfall, solar exposure and moon luminosity on the likelihood of capturing each coat type on a given day. These collected data will establish a baseline for the current distribution of coat types within feral cat populations in Tasmania. Furthermore, this dataset can be revisited in future studies to assess potential changes in these populations, providing insights into whether selective pressure is influencing the morphology of feral cats in this region.

## 2 Materials and Methods

### 2.1 Camera‐Trap Monitoring

The data used in this study were derived from a pre‐existing camera‐trap network established in 2018 for the purpose of wildlife monitoring across Tasmania, Australia. Cuddeback trail cameras (X‐Change Model 1279) were placed on roads, game trails or in naturally open arenas and mounted 30–50 cm from the ground on tree trunks or fallen logs. Camera‐trap sites were, on average, 1013 m apart (standard deviation = 1483 m). Camera traps used a white (strobe) or infrared flash with a passive infrared sensor (being triggered in response to movement and heat). Prior research has demonstrated that flash type does not influence the return rate of feral cats across this camera network (Paton, Buettel, and Brook 2024). No lures were used.

**FIGURE 1:**
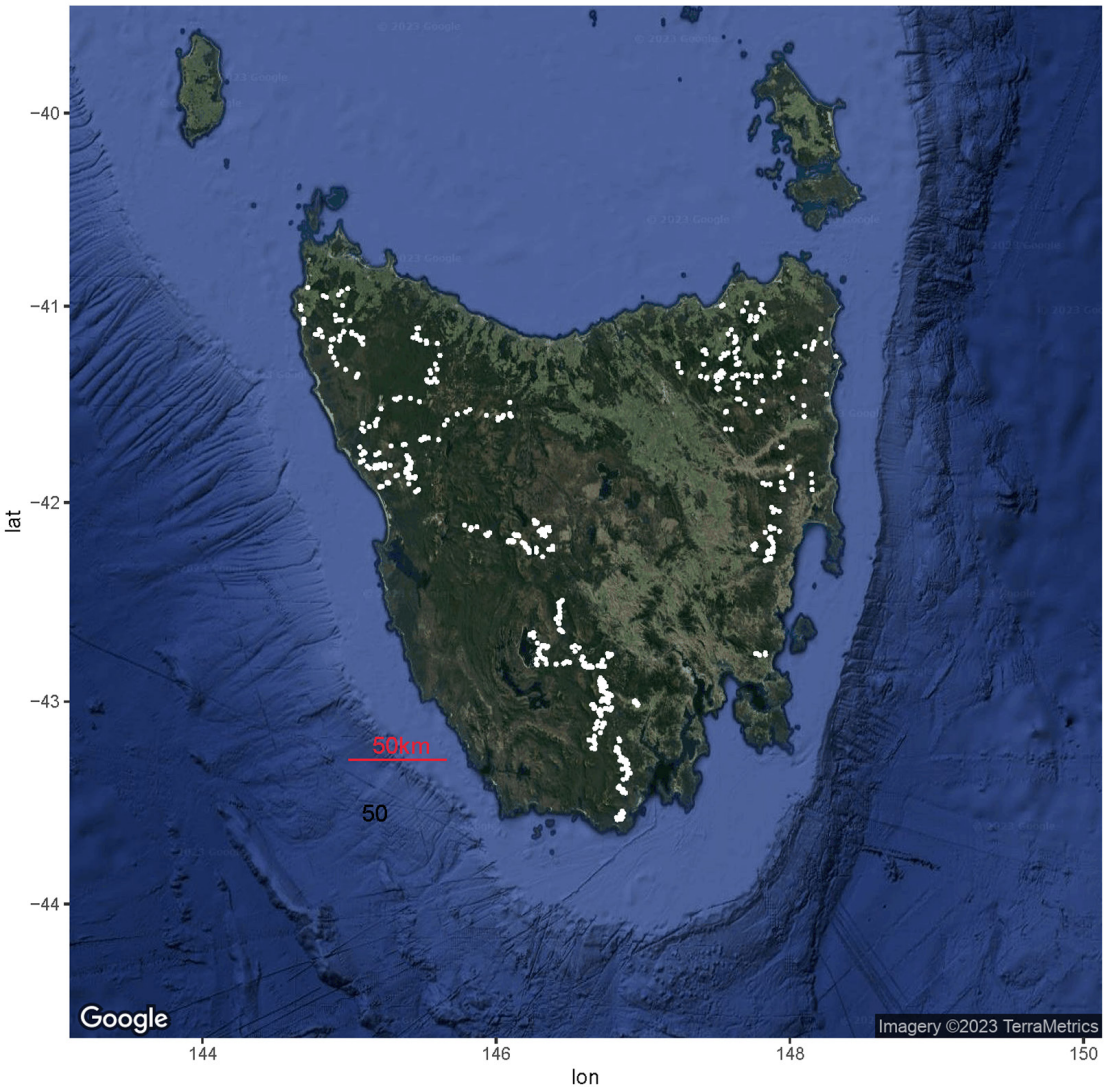
A map of the 651 camera‐trap sites in Tasmania that were used in this study. Each white point represents a camera‐trap site.

We selected only sites where cameras detected cats or operated long enough to have confidence in true absences using the methods of Ringwaldt et al. (2023). This was calculated as an operation time of at least 83 days, the time within which 95% of camera traps recorded a feral cat presence. If a camera operated for < 83 days and returned a feral cat absence, it was considered a potential false negative and discarded. This resulted in 651 camera‐trap sites (Figure 1) and 24,075 images over 234,200 operating days, all of which detected feral cats.

### 2.2 Coat Classification

**FIGURE 2:**
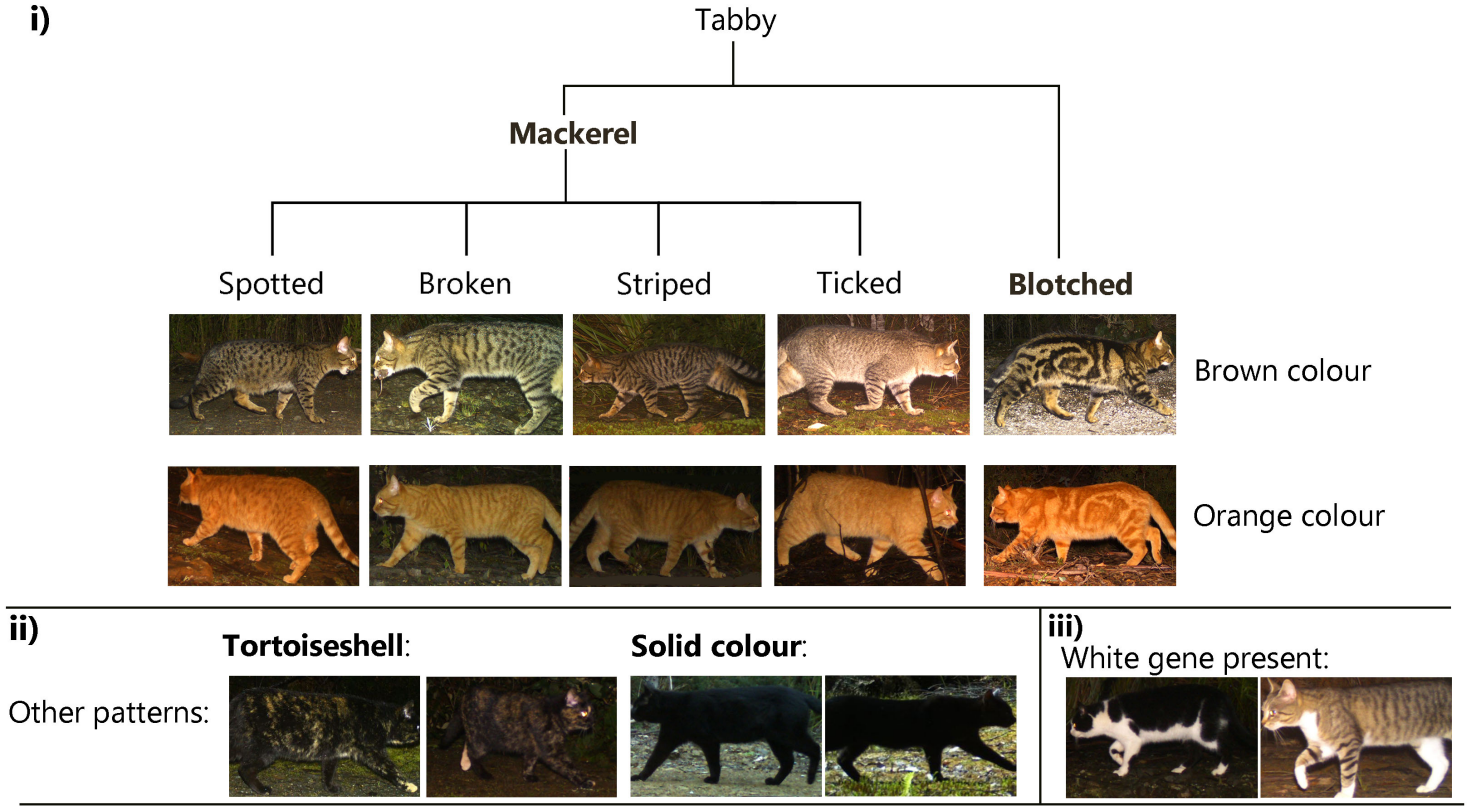
A key describing the variety of feral cat coat types observed in Tasmania, Australia. Tabby pattern types are described within section (i), branching into either blotched or mackerel varieties. All tabby pattern types can be orange or brown depending on the colour gene present. Alternative patterns are displayed in section (ii), with tortoiseshell and solid black cats. Tortoiseshell cats will always display both orange and either brown or black, and solid black cats will display neither orange nor brown. Section (iii) gives two examples of the white gene being present, which can overlap with any of the pattern and colour types described in this key. The patterns written in bold are those used in modelling.

The categories of coat types used were adapted from those described in Eizirik et al. (2010). Each cat image was assigned a pattern and colour (Figure 2). Grey cats were uncommon, and on a gradient to brown, and as such were grouped with brown cats. The presence of white markings is controlled by a separate locus from pattern and colour, meaning white patches could be present on any of the coat types (Kaelin et al. 2011). A tag for ‘white’ was included in addition to colour and pattern to account for this. However, these white patches varied in position in size, often presenting as a small patch on the chest of the cat, on the cat's underbelly or as sock/point colouration. If over half of a black cat's body was white, its pattern was tagged as ‘tuxedo’ rather than black due to the assumed ecological consequences of this colouration. It should be noted that no cats were sighted that were entirely white.

A total of 13 coat types were identified and used to classify cats (Figure 2). These coat types were grouped into five categories of assumed ecological similarity for statistical modelling to overcome the issue of small sample size. These five pattern and colour combinations were solid black, blotched brown, mackerel brown, orange and tortoiseshell (Figure 2). Mackerel included ticked, spotted, striped and broken tabbies, and were grouped due to their overlap in appearance (Figure 2) and due to the similar proposed role in ecology of spots and stripes for camouflage within vegetation (Allen et al. 2011). Blotched brown cats were not grouped with mackerel brown cats as no other felid displays this blotched patterning, and as such its potential ecological role is unknown. Orange mackerel and orange blotched cats were grouped into one category due to the small sample size for each, and the assumption that colouration would have a greater influence on these coat types than pattern. Tuxedo‐patterned cats (i.e., mostly white cats) were excluded from analysis as they were assumed to be ecologically distinct from black cats and too small of a sample to be analysed as an independent pattern type (Table 2). If a cat was obscured from view or if image quality was too poor to distinguish the coat colour or pattern, the coat type was labelled ‘unknown’.

Cats were identified at an individual level using leg, flank and face markings. These were observed by a single researcher, with screenshot ‘snips’ being taken across events (i.e., images separated by more than 1 min between captures) of a suspected individual and compiled in an Excel spreadsheet for comparison. Spreadsheets were divided into regions consisting of clusters of ~50 camera traps with 10 km separation between regions to keep identifications of individual cats manageable. If there was any doubt on the identity of an individual cat, potentially caused by an odd angle, obscured leg and flank or quality image, the cat was labelled ‘unknown’. Identifying cats to an individual level ensured that the pattern and colour assigned to a cat remained consistent despite variations in lighting, camera angle and flash type. Individual identification could not be undertaken for solid black cats as these cats have no distinct markings, resulting in a large proportion of the images having no individual identification. We obtained a rough estimate of the number of individual black cats through the following calculations:

$$\begin{array}{c} \frac{\text{Number of individually identifiable}\;\mathrm{cat}\;\text{images}}{\text{Number of individual cats}}\\ =\text{Number of images}\;\mathrm{per}\;\mathrm{cat} \end{array}$$

$$\frac{\text{Number of black}\;\mathrm{cat}\;\text{images}}{\text{Number of images}\;\mathrm{per}\;\mathrm{cat}}=\text{Estimated number of black cats}$$

This calculation is prone to overestimating the number of black cats in instances where few images or individuals have been captured for other coat types. As such, rather than use the number of individuals in further analyses, we found the ‘modal coat type’ at each camera trap. The ‘modal coat type’ refers to the coat type with the highest number of individual cats captured in a camera trap. This way, even if the number of black cats was overestimated at a site, it is likely that this coat type would have the most individuals regardless. If two or more coat types were tied for the greatest number of individuals, then the coat type with the greatest number of events was selected as the modal coat type, reflecting the idea that this was the ‘dominant’ coat type at a site.

### 2.3 Statistical Analysis

All statistical analyses were undertaken using R v4.3 (R Development Core Team 2010). We summarised the total number of events for each coat type and mapped their presence across Tasmania. An event was defined as a sequence of images of a cat occurring within < 1 min of each other. We examined the influence of spatial features on the presence or absence of feral cat coat types and assessed their effect sizes. Additionally, we assessed how the activity of each coat type differs between days in response to temporal features.

To determine if feral cat presence across spatial features differed between coat types, we used a multinomial logistic regression model (Ripley and Venables 2016). This model contained the modal coat type of a camera‐trap site as the response variable. Solid black cats were used as the reference level, rather than cat absence, as they were the most common and distinct coat type and considering all the selected sites detected feral cats, feral cat absence was not an appropriate reference level. These sites were excluded from further analysis as we were interested in making comparisons between coat types. Six spatial predictors were chosen that were likely to influence colouration (Caro 2005, 2009; Allen et al. 2011; Table 1). These data included climate statistics (Fick and Hijmans 2017), major vegetation groups (Department of Agriculture, Water and the Environment 2018) and topographic roughness (Williams et al. 2010), and distance to nearest town (DTNT). The major vegetation groups were simplified into three broad categories to avoid small sample sizes and overfitting. These were ‘grassland’ which consisted of any grasslands, shrublands, herblands or tussocks, ‘woodland’ which included woodlands with widely spaced trees and open understories and plantations and ‘forest’, which included eucalypt forests and rainforests. Distance to nearest town (DTNT) was calculated for as the number of kilometres between a given camera trap and the centre of the nearest locality and used this as an additional predictor. Locality was defined as a populated township or suburb and sourced from the ArcGIS REST Services Directory, created in 2000.

**TABLE 1:** Spatial predictors examined within a multinomial logistic regression model of five feral cat coat types in Tasmania.

Predictor	Description	Source
Vegetation type	Distributions of vegetation types in Australia in simplified categories: Grassland (shrubland, herblands and tussocks); woodland (open understory and widely spaced trees) and forests (dense eucalypt and rainforests)	National Vegetation Information System, Department of Agriculture, Water and the Environment (2018) (www.environment.gov.au)
Elevation	Elevation above mean sea level, provided in meters	Department of Agricultural Resources (www.data.daff.gov.au)
Topographic roughness	Provides an index of how variable the elevation is within a grid cell	Williams et al. (2010)
Site isothermality	Fluctuations in daily temperature in summer relative to winter	Biodiversity and Climate Change Virtual Laboratory (BCCVL; bccvl.org.au), recorded and summarised from 1976 to 2005
Fraction of photosynthetically absorbed radiation (FPAR)	Average fraction of photosynthetically active radiation absorbed by photosynthetic organisms	Biodiversity and Climate Change Virtual Laboratory (BCCVL; bccvl.org.au), recorded from 2000 to 2014
Distance to nearest town (DTNT)	The number of kilometres between a camera trap and the centre of the nearest locality	ArcGIS REST Services Directory, created in 2000

To examine the influence of temporal features on the activity of each coat type, we used multinomial logistic regression modelling (Ripley and Venables 2016). To minimise noise introduced from unmeasured factors like cloud cover, we subset our dataset to only contain nights that either had > 80% luminosity (full moon) or < 20% luminosity (new moon) using the lunar package (Lazaridis 2022). This resulted in a data frame containing 180,060 camera operating nights, with 89,847 full moon nights and 90,759 new moon nights and a total of 7433 nights with feral cat captures. For each of these dates, we recorded rainfall (in millilitres), solar exposure and minimum temperature from weather stations within 80 km of the camera trap (Bureau of Meteorology, Australia) and used these as predictors within our model. Solar exposure is correlated with cloud cover (McKenzie et al. 1998), and in the absence of data available for cloud cover, we included an interaction effect between solar exposure and moon luminosity. Coat type was used as the response variable in this model. Considering the high number of nights with no cat detections, we added a dummy variable of ‘cat absence’ to use as the reference level.

We calculated the proportion of operating nights a cat event was captured (proportion of dates with events; PDE) on a camera trap as an index for activity of cats. This is a common metric used to compare animal activity between features (Paton, Buettel, and Brook 2024; O'Brien 2011; Bengsen, Butler, and Masters 2012). This was calculated as:

$$\frac{\text{Number of nights with captures}}{\text{Camera operating nights}}\times 100$$

We calculated the mean PDE and standard errors for feral cats on nights with new moons and nights with full moons using the bootstrap method (Canty and Ripley 2017). Considering our data only contained information on whether an image was captured at night or during the day, and the date of capture, we did not attempt further temporal analyses to examine detailed patterns of activity.

## 3 Results

**FIGURE 3:**
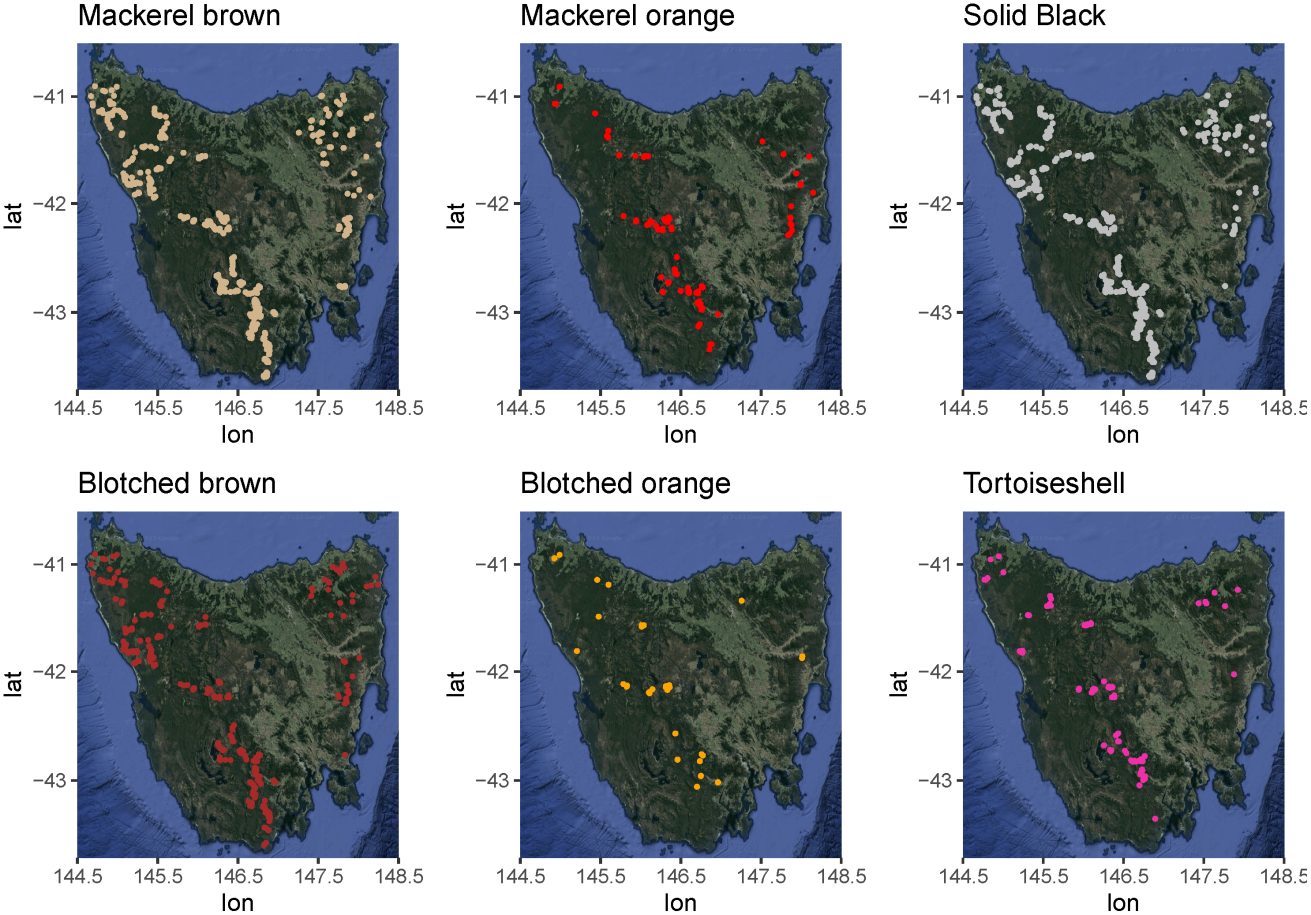
Maps of camera site occurrence for six coat types in the study. Each point on the maps represents a camera‐trap site where the respective coat type was detected. Camera points are not displayed on maps where the specific coat type was not detected during the study period.

**FIGURE 4:**
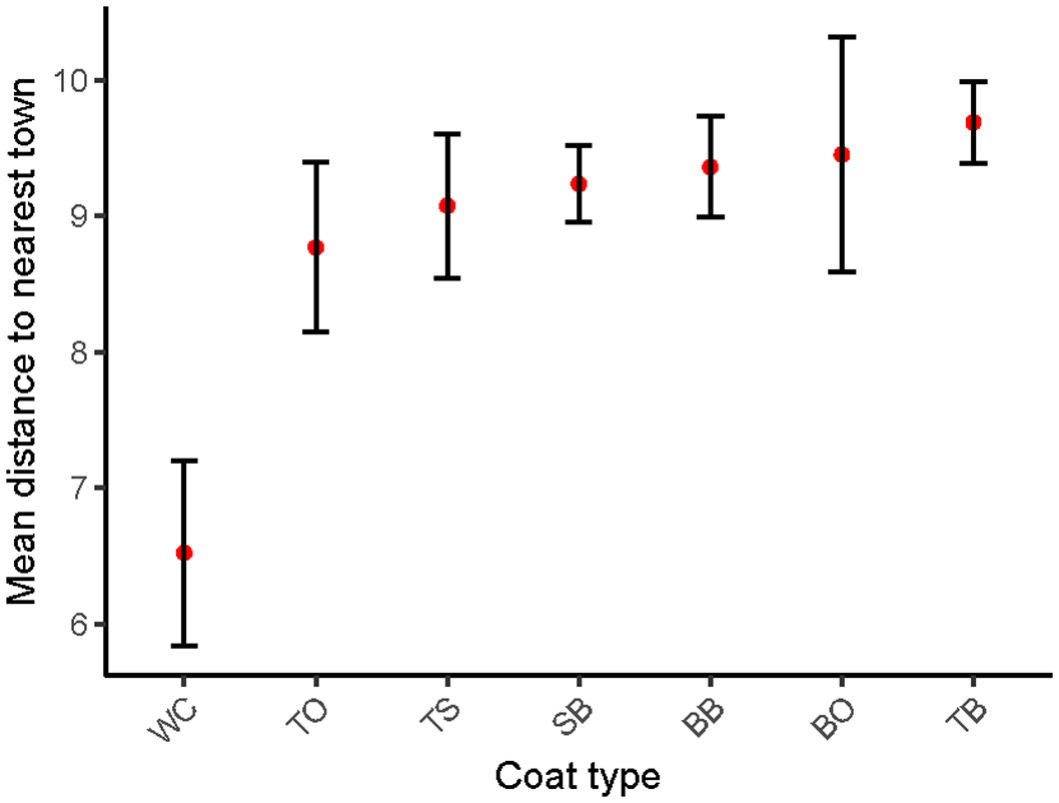
The average distance (km) each coat type was detected to the nearest township. The error bars represent the mean ± the standard error. The coat types listed below are, in order: Any coat with white markings, mackerel orange, tortoiseshell, solid black, blotched orange, blotched orange and mackerel brown.

Of the five major coat categories, solid black cats and mackerel brown cats were the most captured coat types, with 9706 and 7413 events respectively. All five coat types were detected across Tasmania, with the map displaying mackerel orange and blotched orange cats separately to improve resolution (Figure 3). Cats with white markings occurred closer to townships than other coat types (Figure 4) and were captured on 361 images across 49 cameras (Table 2).

**TABLE 2:** A summary of *Felis catus* coat type captures in Tasmania. This includes the number of images captured in the camera‐trap survey for each type, the number of trap nights/days that captured a cat (daily events), the number of individuals identified within each type and the number of cameras that captured images of that coat type. The number of individuals could not be determined for unmarked solid black cats. Unknown cats were those where picture quality or cat angle was too poor to determine pattern, colour or both. Broken, spotted, striped and ticked cats have been grouped into the category of mackerel for both brown and orange colourations and summaries of these are provided in bold. Summary statistics on the subcategories of mackerel brown and mackerel orange cats have been supplied in the ‘subcategories’ column. Counts of events of cats displaying any white patches, stars or point colouration in addition to their assigned pattern and colour are provided in the column ‘images with white’.

Coat type	Subcategories	Image count	Daily events	Percentage of images	Individuals	Images with white	Camera sites
Solid black		9706	8276	40.32	NA	150	478
Blotched brown		3636	3009	15.10	194	38	281
Mackerel brown		7413	6011	30.79	349	41	455
	Broken brown	4266	3545	17.72	204	33	349
	Spotted brown	2878	2264	11.95	129	8	225
	Striped brown	224	167	0.93	11	0	19
	Ticked brown	45	35	0.19	5	0	12
Blotched orange		297	250	1.23	20	14	46
Mackerel orange		761	492	3.16	45	8	87
	Broken orange	457	252	1.90	29	8	62
	Spotted orange	288	229	1.20	12	0	22
	Striped orange	7	6	0.03	3	0	3
	Ticked orange	9	5	0.04	1	0	1
Tortoiseshell		691	508	2.87	55	14	92
Tuxedo		183	160	0.76	11	183	12
Unknown		1388	1014	5.77	NA	0	464
Total		24,075	19,837	100	674	456	651

**FIGURE 5:**
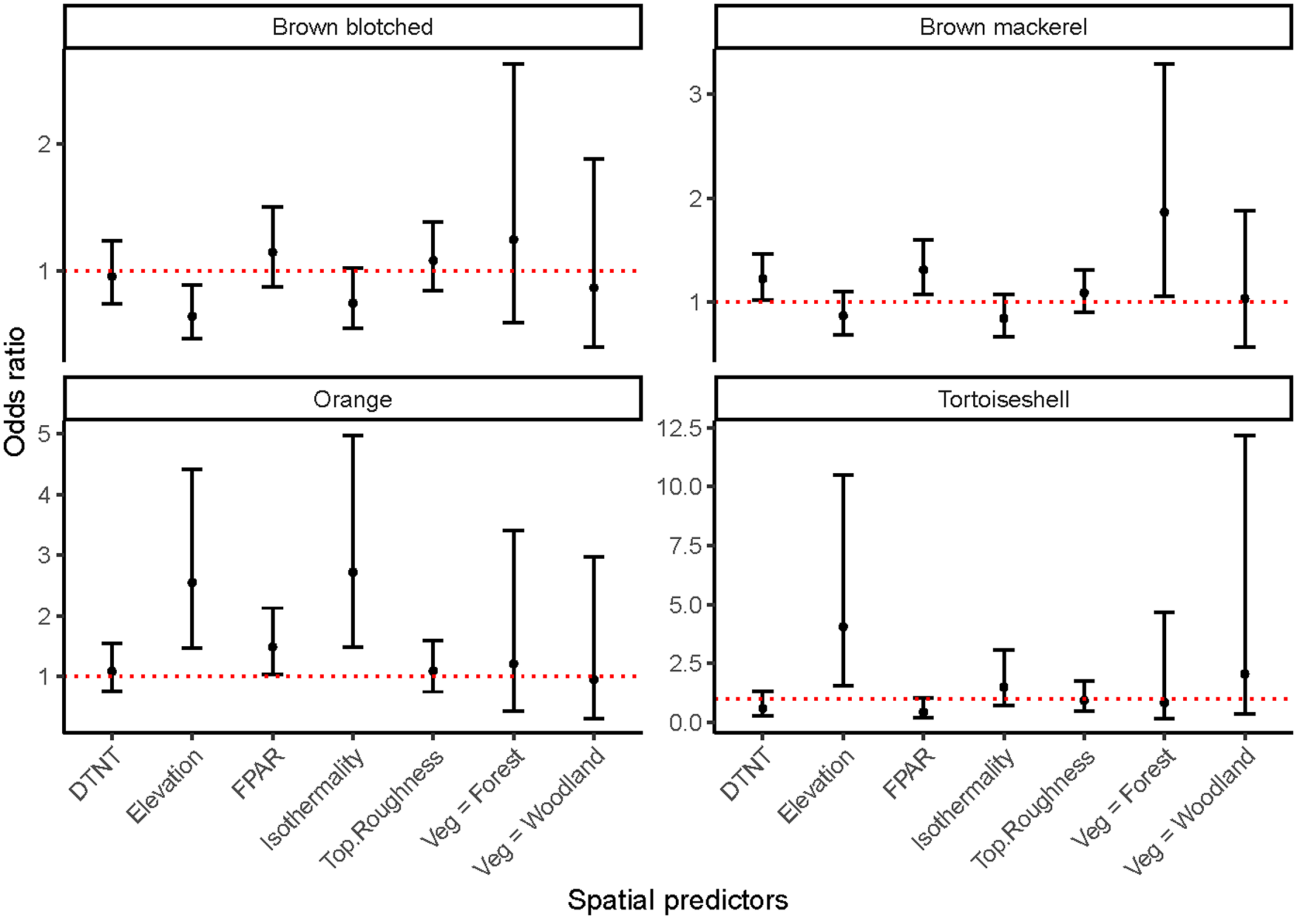
The odds ratios for each predictor from a multinomial regression model of modal coat type (e.g., the coat type with the most individuals present at a site) relative to the presence of solid black cats. The 97.5% confidence intervals are represented by the error bars. The dashed red line intercepts with one, an overlap with which indicates a nonsignificant effect. The predictors on the *x*‐axis are, in order from left to right, rainforest vegetation, topographic roughness, isothermality, distance to nearest town, fraction of photosynthetically absorbed radiation, woodland vegetation, elevation, shrubland vegetation, human‐influenced vegetation and tall eucalypt vegetation. Note that the *y*‐axis for each graph has varying limits.

Elevation, isothermality, FPAR and vegetation type had a significant impact on the modal feral cat coat type within Tasmania. Orange and tortoiseshell cats are more likely to be dominant at higher elevations relative to black cats (odds ratio = 2.5, CI = 1.5, 4.4; odds ratio = 4.0, CI = 1.6, 10.5) (Figure 5). Orange cats were also more likely to be the modal coat type at greater isothermalities than black cats (odds ratio = 2.7, CI = 1.5, 5.0), at sites with a higher FPAR (odds ratio = 1.5, CI = 1.0, 2.1). Blotched brown cats showed a negative relationship with elevation relative to black cats (odds ratio = 0.64, CI = 0.47, 0.89) (Figure 5). Mackerel brown cats were more likely to be the modal coat type in eucalypt forests and rainforests as compared to black cats (odds ratio = 1.9, CI = 1.1, 3.3), as well as sites with a higher FPAR (odds ratio = 1.3, CI = 1.1, 1.6). The remaining spatial features did not influence the dominant coat type of a site (Figure 5).

**FIGURE 6:**
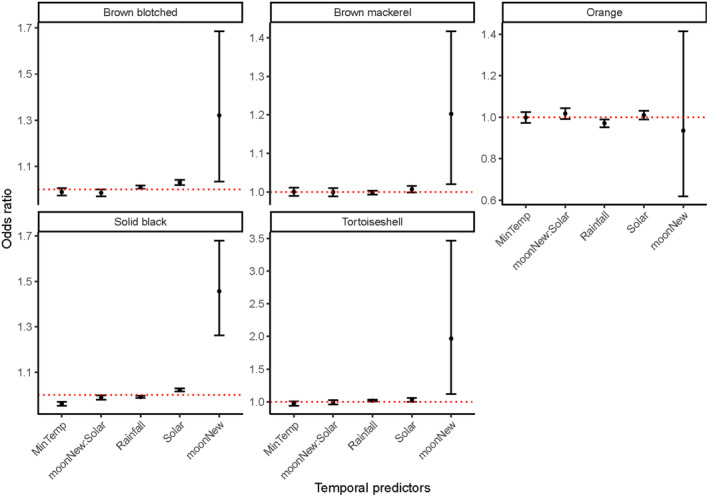
The odds ratios for each of the coat types. In order from left to right, these coat types are blotched brown, orange, solid black, mackerel brown and tortoiseshell. The 97.5% confidence intervals are represented by the error bars. The dashed red line intercepts with one. Odds ratios with confidence intervals that overlap with 1 are assumed to be nonsignificant. The predictors on the *x*‐axis are, in order from left to right, minimum temperature, an interaction effect between moon luminosity and solar exposure, rainfall, solar exposure and moon luminosity. Note that the *y*‐axis for each graph has varying limits.

On average, cats had a PDE of 3.4% (standard error = 0.18%) for nights with a full moon, and 4% (SE = 0.23%) for nights with a new moon. Moon luminosity was an important predictor in capturing all coat types, except for orange cats. All coat types showed an increase in the odds of being captured on new moon nights relative to full moons, except for orange coat types (Figure 6). This effect seemed to be greatest for tortoiseshell cats (odds ratio = 2.0, CI = 1.12, 3.47), though these confidence intervals were wide and the sample size small. The other temporal predictors did not appear important in determining when each coat type would be active (Figure 5).

## 4 Discussion

The melanistic solid black cats and the mackerel brown cats are the most common morphs of feral cats in Tasmania. We found differences in the distribution of the modal coat type across Tasmania with respect to elevation, FPAR, vegetation type and isothermality. Feral cats appear to be more active on new moon nights overall, except for orange cats who were equally likely to be captured on new moon or full moon nights.

There is a relatively stochastic distribution of cat colours and patterns across the state. The positive influence of elevation on orange and tortoiseshell presence may be a result of the lighter colouration of alpine vegetation communities in Tasmania, including button‐grass moors, bolster heath and tussock grasses (Kirkpatrick et al. 1996; Whinam and Chilcott 1999). The brown‐blotched cats showed a negative relationship with elevation. This is the first time the presence of blotched cats has been examined with relation to spatial features, and there is no information in past literature to explain their association with these features. Brown mackerel cats were more likely to be the modal coat type at sites with high FPAR (i.e., dense vegetation) and in eucalypt and rainforests compared to black cats. This may be owing to the additional camouflage provided by their stripey coat pattern breaking up their silhouette within a forested environment (Allen et al. 2011). It could also be interesting to examine the influence of climate on coat colour and pattern across more variable habitats. For instance, lighter fur colours, like orange, may be expected in arid environments, where it could reflect light to aid in thermoregulation (Caro 2005). Future studies could compare across other cat surveys in Australia and see if the trends we observed were consistent, and to examine coat types across a greater range of habitats and conditions.

Camouflage is the major driver of coat evolution in other felid species, as it increases hunt success for these stalk‐and‐ambush predators (Allen et al. 2011). However, in Australia, many native prey species are predator naïve (Moseby et al. 2015). In addition, at least some marsupial species in Australia have dichromatic vision (Hemmi 1999; Deeb 2010), and as such would struggle to distinguish between orange and brown colouration in a feral cat, though the abundance of dichromatic and trichromatic vision in marsupials is still under research (Ebeling, Natoli, and Hemmi 2010). This might diminish the advantage of camouflage and, consequently, the selective pressure on feral cats, particularly in an environment with minimal predatory competition. Comparisons between distinctly different habitats, such as deserts, dry arid forests or tropical rainforests, would reveal clearer phenotypic variations between cat populations.

Tasmanian feral cat populations exhibit both patterned morphs and solid black individuals, superficially resembling other felid species that contain these phenotypes due to disruptive selection (Graipel et al. 2014). Contrary to expectations set by these other felid species, we observed that nearly all coat types are more active on dark nights. This suggests that the diverse phenotypes observed in our feral cat populations result from the founding populations' array of coat types rather than disruptive selection. Prey species of mammal in south‐eastern Australia have been shown to be more active on dark nights (Linley et al. 2020). Additionally, larger species of felid have been identified as having the highest rate of hunting success on dark nights (Funston, Mills, and Biggs 2001). As such, the higher activity of these cats on dark nights may be owing to the higher prey activity, with the darkness providing camouflage, and improving potential hunt success.

No difference was found in the activity of orange cats across moon luminosities. This is surprising, as one would assume brightly coloured morphs intuitively benefit from hunting on dark nights (Caro 2005; Coss, Ramakrishnan, and Schank 2005; Graipel et al. 2014). The consistent activity of orange cats across full moon and new moon nights may be a result of aggressive males patrolling territory or seeking queens, rather than hunting. In domestic populations, around 80% of orange cats are male due to the sex‐linked nature of the colouration gene (González‐Ramírez and Landero‐Hernández 2022). Most orange cats were also male in populations across south‐east Australia (Jones and Horton 1984; Read and Bowen 2001). Orange male cats are consistently heavier than other male cat coat types by 200–300 g (Jones and Horton 1984; Pontier, Rioux, and Heizmann 1995). Liberg (1983) showed that in farm cats, the heaviest males were the most dominant and aggressive. Perhaps this trend of aggressive behaviour in large, orange males persists in Tasmania. We would require a larger sample size of orange individuals to validate these ideas, or GPS collar tracking of a variety of coat types. There is the potential for these data to be gleaned from past studies that used GPS trackers as well. There is potential for seasonality to influence when and where male cats roam, with male cats in New Zealand being less active during summer months (Langham 1992). Future studies examining this point should also consider the potential for seasonal effects on male cat activity. No other temporal features influenced the probability of detecting a certain coat type on a given night. The weather stations used in our study were often tens of kilometres from the nearest camera trap, which may have limited the inferences that could be made from these data.

The distribution of coat types in our study did not appear to be largely influenced by the predictors we examined. Instead, the frequencies and distribution of coat types may be reflective of those within the founding populations of feral cats across Tasmania. For example, black cats may have been genetically dominant within the early introductions of feral cats to Tasmania. While data on founder populations are unavailable, we can explore the limited data we have on current coat colour and pattern frequencies across Tasmania's domestic cats. A study examining the characteristics of 4736 shelter cats within the Tasmanian Royal Society for the Prevention of Cruelty to Animals (RSPCA) between 2006 and 2010 found that ‘tabby’ and black cats were the most common coat types within shelter populations (36% and 31% respectively), followed by tortoiseshell (10%), and ‘other’ (23%) (Alberthsen et al. 2016). In that study, tabby encompassed brown mackerel and blotched cats (Alberthsen et al. 2016). If we assume individuals across coat types have similar rates of capture, we can see that black cats are more prevalent in Tasmania's feral populations than in the sampled shelter populations comprising stray and domestic cats. Considering that black coat colouration is recessive to mackerel or tabby morphs (Eizirik et al. 2010), if we were to assume that feral populations are a result of immigration from domestic cats, the prevalence of this colouration could indicate that black cats are more successful in feral populations compared to brown mackerel cats. This idea could be re‐examined in future surveys of feral cats where adjacent domestic cat populations are better understood. Alternatively, the founding populations of cats in Tasmania may have consisted of a majority of black cats, leading to their prevalence in wild populations.

White cats were rare within the feral populations and consistently present closer to townships than the other coat types surveyed. Dorn, Taylor, and Schneider (1971) monitored ‘free‐roaming’ domestic cats and indoor cats. Within the free‐roaming group, they examined 34 cats with white coats and 49 cats without white. They found that white cats had 13.4 times greater risk of developing cutaneous squamous cell carcinomas (skin cancer) than all other cats combined. As such, the reduction in the presence of cats with white markings with distance from township could be evidence of a strong selective pressure against these cats. It would be worthwhile for future research to compare the frequency of the appearance of white patches in nearby domestic populations to further inform if the absence of white in our survey was due to selective pressure.

Solid black cats have no discernible markings, and therefore cannot be identified to an individual level. This can be problematic when undertaking any modelling that requires individual identification (e.g., spatial‐mark‐resight, minimum‐known alive). These models assume that marked cats are a random sample of the population with the same movement ecology as unmarked cats (Efford and Hunter 2018). Our findings broadly support this assumption, whereby despite their high prevalence, unmarked cats appeared to occur in the same habitats as marked individuals. If unmarked or unidentifiable captures are too high, abundance and density modelling cannot be undertaken (Sparkes et al. 2021). What is ‘too high’ will depend on the sample size and recaptures of a given study, though over 50% of images being unidentifiable can affect model accuracy (McClintock 2011). For example, Sparkes et al. (2021) found that around 70% of feral cat images across several surveys were unidentifiable, and as such was unable to undertake density estimates. Researcher's wishing to model cat density and abundance in Tasmania should be aware of the high proportion of unmarked cats present.

## 5 Conclusion

Our study has provided the first comprehensive overview of coat colour and pattern distributions for feral cats in Tasmania, Australia. While spatial features did not strongly dictate coat type distribution, temporal analysis showed a consistent preference for new moon nights among all coat types, with orange cats exhibiting consistent activity levels across moon phases, potentially linked to territorial behaviours. These findings suggest that selective pressures on feral cat coat types in Tasmania may be limited or influenced by factors beyond immediate environmental characteristics. Future studies should explore these dynamics further, comparing feral cat populations with adjacent domestic groups and examining diverse habitat types to gain a comprehensive understanding of the ecological drivers shaping coat morphology in Australian environments.

## Author Contributions

**Alexandra J. Paton:** conceptualization (lead), formal analysis (lead), investigation (equal), methodology (equal), project administration (lead), visualization (lead), writing – original draft (lead). **Barry W. Brook:** data curation (equal), formal analysis (supporting), supervision (supporting), writing – review and editing (supporting). **Jessie C. Buettel:** conceptualization (supporting), data curation (equal), supervision (lead), writing – review and editing (lead).

## Conflicts of Interest

The authors declare no conflicts of interest.

## Data Availability

Datasets and analyses are available at: https://github.com/AlexJPaton/CoatChapter.
